# 16 Ch × 200 GHz DWDM-Passive Optical Fiber Sensor Network Based on a Power Measurement Method for Water-Level Monitoring of the Spent Fuel Pool in a Nuclear Power Plant

**DOI:** 10.3390/s21124055

**Published:** 2021-06-12

**Authors:** Hoon-Keun Lee, Jaeyul Choo, Joonyoung Kim

**Affiliations:** 1 Department of Safety Research, Korea Institute of Nuclear Safety, 62 Gwahak-ro, Yuseong-gu, Daejeon 34142, Korea; hklee@kins.re.kr; 2 Department of Electronics Engineering, Andong National University, 1375 Gyengdong-ro, Andong-si 36729, Korea; jychoo@anu.ac.kr; 3 Department of Smart Information and Communication Engineering, Sangmyung University, 31 Sangmyungdae-gil, Dongnam-gu, Cheonan-si 31066, Korea

**Keywords:** remote passive sensing, power measurement method, optical fiber sensor network, dense wavelength division multiplexing (DWDM), water-level monitoring

## Abstract

This paper presents a remote 16 Ch × 200 GHz dense wavelength division multiplexing (DWDM)-passive optical fiber sensor (OFS) network. We particularly investigate the remote water-level monitoring capability of the OFS network based on an optical power measurement that features simplicity and a fast processing speed. The OFS network utilizes a seeded amplified spontaneous emission (ASE) light that is spectrum-sliced and distributed by an arrayed waveguide grating (AWG) towards multiple sensing units (SU), where each SU is installed at a different height in the water pool. Then, each SU reflects either of the two different optical powers according to the medium (air vs. water) back to the monitoring station. Therefore, the total received optical power at the monitoring station linearly changes according to the water level. We can simply recognize the water level by utilizing the optical power meter (OPM) at the monitoring station rather than the optical spectrum analyzer (OSA), which is bulky and expensive and requires a relatively long processing time. Consequently, we can reduce the system complexity, processing time, and cost (both installation and maintenance). However, the OPM-based OFS network requires a new methodology to derive the water level from the measured optical power. Thus, we come up with the reference-to-power ratio (RPR) analysis, which can be used for the maximum distance analysis as well as water level recognition. Based on the new reception architecture supported by the new post-processing scheme, the OFS network can distinguish 17 different water levels of the SFP at the monitoring station, which is >40 km away from the SFP, without using any active devices (such as optical amplifiers) at the remote places.

## 1. Introduction

Water is a crucial resource to nuclear power plants (NPPs), especially for the pressurized water reactor. For example, the water inside the spent (or storage) fuel pool (SFP) of NPPs not only cools down the decay heat but also prevents any possible leaks of radiation fields [1,2]. Thus, information regarding the water (such as level, temperature, radiation dose level, etc.) must be provided to the main control room (or emergency operation facility) even after the physical damages caused by various natural hazards such as tsunami, earthquakes, etc. As we observed in the Daiichi NPP accident of Fukushima, however, such catastrophic events severely impair the water management (e.g., monitoring) system, especially due to the loss of electric power [3]. Subsequently, the Nuclear Regulatory Commission (NRC) issued an order to require all the United State’s NPPs to install water level instrumentation in their SFPs with three distinct water levels that could be remotely reported [4]. This indicates there are new needs for technologies to remotely monitor the water status without electrical power supplied to the local field for surviving a variety of harsh environments caused by high temperature and radiations. In response to this, a remote-sensing network employing optical fiber sensors (OFS) has been proposed in recent years [2,5,6,7]. The OFS network has been drawing increasing attention due to various advantages such as passive/remote-sensing features, and excellent tolerance towards electromagnetic inferences and radiations. Moreover, it can be readily applied to many different industrial fields, including hot spot monitoring for electrical power cables, external intrusion, railway monitoring, structural health monitoring, etc. [8,9,10,11].

The OFS techniques can be classified into two groups according to the measurement principle: (i) distributed sensors and (ii) discrete sensors. The distributed sensors are typically realized by Rayleigh-, Brillouin-, or Raman-scattering-based optical time-domain reflectometer (OTDR), enabling continuous monitoring, i.e., high spatial resolutions within a specific area/range [12,13,14,15]. However, such high resolution is achieved at the expense of the measurement range [16,17]. On the other hand, the discrete level sensors utilize multiple sensing units (SU) that are installed, e.g., inside the water pool to capture the environmental data, where the SUs can be implemented with optical filters (e.g., Fabry–Perot cavities [18,19,20] and Bragg grating [21,22,23]) or optical reflectors (e.g., optical fiber tips [6,7,24,25]). The use of multiple SUs requires optical (or electrical) multiplexing techniques [26,27,28], and thus would result in limited resolution.

We recently proposed and demonstrated a simple OFS network offering a good mixture of sensing distance (>km) and resolutions (e.g., <tens of cm) [29,30]. Specifically, we use the amplified spontaneous emission (ASE) light, where its optical spectrum is encoded according to the water level of the SFP. For this, the system needs to include the dense wavelength division multiplexing (DWDM) filter such as an arrayed waveguide grating (AWG) that inherently offers passive, self-referencing characteristics and low insertion losses. Subsequently, it allows for simple architecture and an intuitive analysis process with robustness to external temperature changes. Moreover, the distance between the monitoring station and target can be extended up to tens of kilometers (e.g., 40 km) once the optical backscattering at fiber-optic cables is properly managed [30]. However, this scheme needs to sweep the whole spectrum of the ASE light as broad as tens of nm throughout, e.g., the telecom C-band (1530–1565 nm). It is basically performed by an optical spectrum analyzer (OSA), which is costly and bulky. Additionally, sweeping the broad spectral band is a time-consuming process that makes agile reaction difficult. It will be even more challenging as the number of SUs (i.e., optical bandwidth) increases (e.g., enhanced sensing resolution). As an alternative to the OSA, one can consider the use of an optical power meter (OPM) that consists of a simple optoelectronic device (e.g., p-i-n photodetector) and electrical amplifiers [31]. Then, the OPM detects the total power of the optical signals that come from multiple SUs, radically reducing the processing time (e.g., sub-milliseconds), complexity, and thus the total cost of ownership (TCO). The replacement of instruments (from OSA to OPM) will raise some concerns, though. First, the sensing distance of > km may not be achievable due to the Rayleigh backscattering (RBS) in fiber-optic cables. Secondly, new referencing and post-processing methodologies are necessary.

In this paper, we fully demonstrate the OPM-based passive OFS network that utilizes the DWDM grid. To be specific, we investigate remote-sensing performances with reference-to-power ratio (RPR) analysis that offers decision criteria. For this, we re-design the monitoring station so that the water level can be determined in comparison to the received optical power and reference power. In addition, we investigate the impacts of RBS on the system performance as a function of transmission length via theoretical analysis as well as experiments. Ultimately, we apply the new system design to the dual-path configuration to show that the distance can be increased >8 times simply by eliminating the RBS limitations without any optical amplifiers.

## 2. Architecture of DWDM-Passive OFS Network Based on Optical Power Measurement

**Figure 1:**
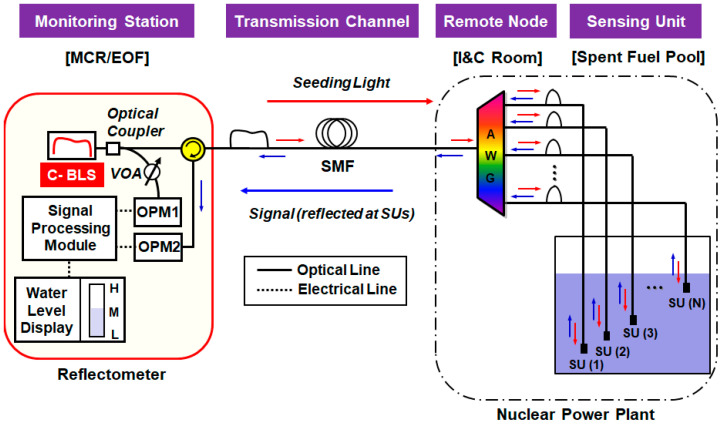
Architecture of the dense wavelength division multiplexing (DWDM)-passive optical fiber sensor network based on single-path configuration. VOA: Variable Optical Attenuator, SU: Sensing Unit, MCR: Main Control Room, EOF: Emergency Operation Facility.

The optical-power-measuring OFS system comprises four function blocks: (i) monitoring station (Main Control Room or Emergency Operation Facility); (ii) transmission channel (single-mode fiber, SMF); (iii) remote node (Instrumentation and Control room); and (iv) multiple SUs in the SFP, as Figure 1 illustrates [32]. The monitoring station has transmitting and receiving parts separated by the optical circulator, forming a reflectometer. The transmitting part seeds ASE light into the network for providing (i) SUs with optical power and (ii) the receiving part with reference power via a 1 × 2 optical coupler. Then, one of two OPMs at the receiving part (OPM1) measures the reference power while the other (OPM2) measures the signal power. The variable optical attenuator (VOA) was inserted between the optical coupler and OPM1 to adjust the level of reference power. The AWG at the remote node distributes the spectrum-sliced ASE light towards the SUs, and combines the signals from multiple SUs, where each SU represents a specific water level. Each SU is a fiber-optic connector where a small portion of the spectrum-sliced ASE light is back-reflected (due to the Fresnel reflection) towards the monitoring station. The reflectance of SU depends on the refractive index of the medium in which the SU is submerged (i.e., the water or the air). Thus, the measured signal power by the OPM2 will linearly change according to the water level, which is compared to the reference power (measured by OPM1) in order to estimate the actual height. It is worth noting that the transmitting and receiving parts share a single SMF for optical transmission, so-called a single-path configuration network. Otherwise, a dual-path OFS network utilizes two separate transmission paths for transmitting and receiving optical signals. Those remote-sensing performances according to the network configuration will be discussed in Section 4.

For the quasi-distributed (discrete) water-level monitoring system, one of the main drawbacks is its limited multiplexing capability. The proposed DWDM-based OFS network can provide a strong advantage compared to other various multiplexing techniques in terms of channel scalability. The channel capacity can be easily increased by utilizing another wavelength band of BLS with a cyclic characteristic of AWG [33] and/or reducing the channel bandwidth of AWG [34]. Moreover, it can provide a very simple architecture with a self-referencing function for the determination of water level. To compare with other multiplexed passive OFS networks for water-level monitoring, we summarized the related state-of-the-art in Table 1.

## 3. Water Level Measurement—Principle and Results

### 3.1. Operating Principle

In this section, we describe Fresnel reflection, the basic operation principle of our system. The change in the surrounding medium of SUs induces the change of reflected optical power, where the Fresnel coefficients ($R_{a}$ for the air and $R_{w}$ for the water) are represented as [35]:

(1) $$R_{a}={\left(\frac{n_{f}-n_{a}}{n_{f}+n_{a}}\right)}^{2},\;R_{w}={\left(\frac{n_{f}-n_{w}}{n_{f}+n_{w}}\right)}^{2}$$

where $n_{f}$, $n_{a}$, and $n_{w}$ are the refractive indices of the optical fiber, the air, and the water, respectively. The approximate values of $n_{f}$, $n_{a}$, and $n_{w}$ are 1.449, 1.000, and 1.315, respectively, when the ambient temperature is 10 ℃ [31]. We suppose these values are constant as they hardly change for the wavelength and temperature within the C-band. Then, the Fresnel reflections $R_{a}$ and $R_{w}$ are −26.3 dB (0.23%) and −14.7 dB (3.36%), respectively, having a power ratio (e.g., $R_{a}-R_{w}$) of 11.6 dB. However, the fiber-optic system of the real world always has unwanted back-reflections and optical crosstalks that are generated by passive devices, connectors, fiber splice points, etc. [30]. This is also detected by the receiver (OPM2 in Figure 1) being background noise, and thus, reduces the power ratio.

Then, the received optical power (measured by OPM2) at each channel can be expressed as Equations (2) and (3) without taking RBS effects into consideration:

(2) $$P_{a}\;\left[W\right]\;=\;\int_{\lambda }^{\lambda +\Delta \lambda_{ch}}{\left|E_{ASE}\left(\lambda \right)\right|}^{2}\;\cdot \;\left[{\left|\sqrt{T_{AWG}\left(\lambda \right)}\right|}^{4}\;\cdot \;R_{a}\;\cdot \;10^{\left(-\frac{L_{IL}}{10}\right)}+10^{\left(\frac{BN}{10}\right)}\right]d\lambda$$

(3) $$P_{w}\;\left[W\right]\;=\;\int_{\lambda }^{\lambda +\Delta \lambda_{ch}}{\left|E_{ASE}\left(\lambda \right)\right|}^{2}\;\cdot \;\left[{\left|\sqrt{T_{AWG}\left(\lambda \right)}\right|}^{4}\;\cdot \;R_{w}\;\cdot \;10^{\left(-\frac{L_{IL}}{10}\right)}+10^{\left(\frac{BN}{10}\right)}\right]d\lambda$$

where $P_{a}$ and $P_{w}$ represent the received optical power of a single channel ($\lambda \;\sim \;\lambda +\Delta \lambda_{ch}$) when the SU is in the air and in the water, respectively. $E_{ASE}\left(\lambda \right)$, $T_{AWG}\left(\lambda \right)$, $L_{\mathrm{IL}}$, and BN correspond to the electrical field of ASE, AWG transfer function, total insertion loss, and background noise power of the received optical signal, respectively. The ASE light can be modeled as uniformly spaced spectral components that have a constant amplitude and uniformly distributed random phase within [0~2π]. Each channel of AWG can be considered as a band-pass filter that has a Gaussian-shape passband at the center wavelength of $\lambda_{c}$, as represented in Equations (4) and (5) [36]:

(4) $$T_{AWG}\left(\lambda \right)\;=\;exp\left[-ln2{\left(\frac{\lambda -\lambda_{c}}{\Delta \lambda_{BW}/2}\right)}^{2m}\right]$$

(5) $$\lambda_{c}=\lambda_{1}+\left(n-1\right)\;\cdot \;\Delta \lambda_{ch}$$

where $\Delta \lambda_{BW}$ and m correspond to the 3 dB bandwidth of each AWG channel and the order of filter, respectively. In addition, $\lambda_{1}$ is the center wavelength of the first channel, $\Delta \lambda_{ch}$ the channel spacing, and n the total number of channels. In simulation, we used m = 1.35, $\Delta \lambda_{BW}$ = 1.03 nm, and $\Delta \lambda_{ch}$ = 1.6 nm. The insertion loss ($L_{IL}$) in Equations (2) and (3) indicates total optical attenuation throughout the whole signal path, which can be expressed in dB scale as below:

(6) $$L_{IL}\;[\mathrm{dB}]=2\left(L_{OC}+L_{AWG}+L_{SU}+L_{SMF}\right)$$

where $L_{OC}$, $L_{AWG}$, $L_{SU}$, and $L_{SMF}$ represent insertion losses of the optical circulator, AWG, SU, and SMF, respectively. The factor of 2 on the right-hand side means that the system has a loop-back structure. Insertion/splitting losses of the 1 × 2 optical coupler for the provision of reference power level is not included, for simplicity. The last term of the right-hand side in Equations (2) and (3) represent the background noise coefficient (BN), which is defined by a ratio of the received optical noise to the ASE power injected into the SMF via optical circulator.

**Figure 2:**
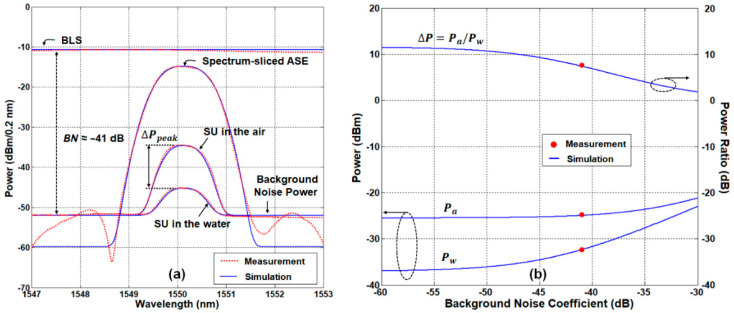
Fresnel reflections in the air and water. (**a**) Measured and simulated optical spectra for single channel. (**b**) Fresnel reflection power ratio ($\Delta P=P_{a}/P_{w}$) according to the background noise coefficient.

We verified the theory via experiment as well as simulation. Note that we used LC/PC type fiber-optic connectors as SUs in order to reduce the footprints as well as the surface tension. In the simulation, $L_{OC}$, $L_{AWG}$, $L_{SU}$, $L_{SMF}$, and BN were 1.5 dB, 4.5 dB, 1 dB, 0.22 dB/km, and −41 dB, respectively. Figure 2a shows the measured/simulated optical spectrum of the spectrum-sliced ASE, and the back-reflected optical signal (by the SU) captured with the OSA at the monitoring station. The power ratio of two optical signals at the center wavelength *(*$\Delta P_{peak}$) was about 10.6 dB, which is 1 dB smaller than the prediction using Equation (1) due to BN. We then investigate the impact of BN on the optical power ratio ($\Delta P=P_{a}/P_{w}$), as shown in Figure 2b. For this, we utilized OPM2 at the receiving part of the monitoring station. In the simulation, we used Equations (2) and (3). Both $P_{a}$ and $P_{w}$ increase with the background noise coefficient (BN). Since $P_{w}$ is smaller than $P_{a}$; however, the impacts of BN are more significant for $P_{w}.$ As a result, the power ratio (ΔP) decreases as BN increases. In the experiment, ΔP was measured to be ~7.6 dB.

### 3.2. Water Level Measurement Results

Optical signals from the multiple SUs are added up by the AWG, as Figure 1 shows. Substituting Equations (1), (4)–(6) into (2) and (3), the total optical power ($P_{Signal}$ in Watt) can be written as:

(7) $$P_{signal}\left(i\right)\;=\;\int_{\lambda }^{\lambda +\Delta \lambda_{ch}}{\left|E_{ASE}\left(\lambda \right)\right|}^{2}\;\cdot \;\left[{\left|\sqrt{T_{AWG}\left(\lambda \right)}\right|}^{4}\;\cdot \;\gamma \left(i\right)\;\cdot \;10^{\left(-\frac{L_{IL}}{10}\right)}+10^{\left(\frac{BN}{10}\right)}\right]d\lambda$$

(8) $$\gamma \left(i\right)\;=\;\left[i\;\cdot \;R_{w}\;+\;\left(N-i\right)\;\cdot \;R_{a}\right]$$

where $\gamma \left(i\right)$ represents the total reflection coefficient. In Equation (8), i and N mean the water level (i.e., the number of SUs in the water) and the total number of SUs deployed in the SFP, respectively. Thus, $\left(N-i\right)$ in Equation (8) becomes the number of SUs exposed in the air.

**Figure 3:**
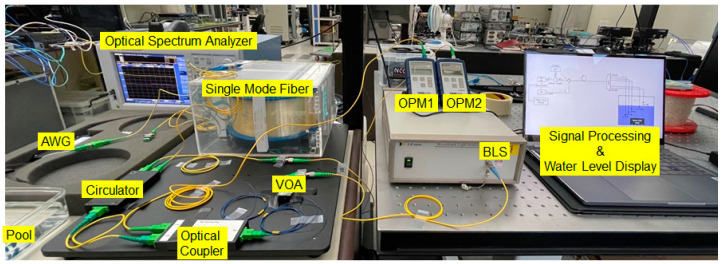
Experimental setup for demonstration of the dense wavelength division multiplexing (DWDM)-passive optical fiber sensor network based on single-path configuration.

We experimentally demonstrated the OFS network in a back-to-back condition (i.e., using 2 m long fiber-optic patch-cord instead of the SMF spool), and compared the results with theoretical predictions (i.e., $P_{Signal}$). Figure 3 shows the setup we built with the off-the-shelf components for the proof-of-concept experiment. The setup includes the C-band BLS (OFB-ACB, LiComm) that generates ASE light (bandwidth > 32 nm and flatness < 1.5 dB). To minimize optical attenuation, we used the 99:1 optical coupler that induces ~1 dB loss to the seed light and 20.3 dB loss to the reference light. In addition, the optical circulator induces 0.8 dB insertion loss to the seeded light. Consequently, the optical power of ASE light at the input of AWG was about 14.1 dBm. The flat-top AWG (ANDevices) comprised 16 channels (i.e., 16 SUs), where each channel spacing and 3-dB bandwidth were 1.6 nm and 1.03 nm, respectively. In addition, the insertion loss of the AWG was <3.5 dB which is much lower than that of optical splitters [28]. The 16 standard LC/PC output connectors of the AWG (i.e., SUs) were placed in the water pool. The use of 16 SUs allows the measuring of 17 discrete water levels (i.e., from step 0 to step 16). In the monitoring station, we utilized OPMs (PM20, Thorlabs) that are able to measure the optical power from 1 nW to 20 mW at the C-band.

**Figure 4:**
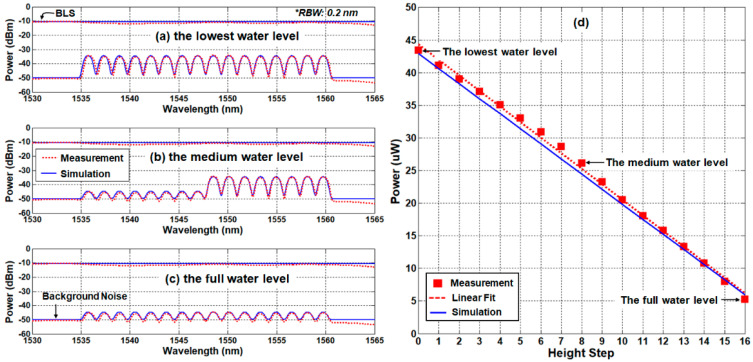
Simulation and measurement results according to the water level in the back-to-back condition: (**a**) the spectra of lowest water level, (**b**) the spectra of 1/2 water level, (**c**) the spectra of full water level, and (**d**) the received optical power as a function of the water level.

Figure 4a–c shows the optical spectrum for three different water levels: (a) empty, (b) half-full, and (c) full SFPs. The spectral peak power ratio ($\Delta P_{peak}$) of 10.6 dB was maintained in all cases. Figure 4d shows the received optical power at OPM2 (i.e., $P_{signal}$) as a function of water level (i.e., the number of SUs in the water). The experimental results show a good agreement with the theoretical estimations. Specifically, the optical power decreases as water level increases, as Equation (7) describes, where the largest mismatch between the experimental and analytical results was less than 3.6%. This is attributed to channel-dependent factors such as the insertion losses of AWG, SUs, and the spectral variation of the BLS power. Such factors eventually affect the linear characteristic of the sensor system. Thus, the components should be carefully selected in the design process to guarantee linearity. We investigate the sensor’s linearity by drawing the fitting curve ($y=b_{1}x+b_{0}$, see the dashed red line) throughout the experimental data. The linear curve has the slope ($b_{1}$) of −2.387 and the intercept point ($b_{0}$) of 44.348, respectively. The coefficient of determination ($R^{2}$) for the linear fit is 0.997, which shows an excellent linear response.

### 3.3. System Performance Evaluation with the Reference-to-Power Ratio

We discussed in Section 3.2 that the channel-dependent power/loss variations cause the system nonlinearity. In addition, environmental (e.g., temperature) variations during the operation cause the index change of the water [31]. It eventually will result in the change of the optical power reflected by the SUs (i.e., 1.9% change for the temperature of 10–100 ℃ in the full water-pool case). Thus, the optical power measured by OPM2 will be $P_{OPM2}=P_{signal}\left(i\right)\;+\;\Delta P_{i}$, where $\Delta P_{i}$ represents the power deviation due to the degradation factors. As such, if $\left|\Delta P_{i}\right|\;>\;\left|\left(P_{signal}\left(0\right)\;-\;P_{signal}\left(N\right)\right)/N\right|$, the water level will be misread in the monitoring station.

To evaluate the system performance, we utilize a reference-to-power ratio (RPR), which is defined as:

(9) $$RPR\left(i\right)\;\left[\%\right]\;=\;\frac{P_{signal}\left(i\right)\;+\;\Delta P_{i}}{P_{ref}}\times 100,\;\;\;\;\;\;\;i=0,\;1,\;2,\;\cdots ,N.$$

where $P_{ref}$ represents the reference optical power measured by OPM1. For the sake of system reliability, periodic calibrations are necessary. In the calibration process, we preset $P_{ref}$ to be the same as $P_{signal}\left(0\right)$ with using the VOA as shown in Figure 1. Then, $P_{\mathrm{OPM}2}$ is divided by $P_{\mathrm{ref}}$ in the post-process stage to calculate $RPR\left(i\right)$. The RPR difference between the estimated power ($P_{signal}$) and actually received power ($P_{signal}+\Delta P_{i}$) are: $\Delta RPR\left(i\right)\;=\;\Delta P_{i}/P_{ref}$. Then, $\Delta RPR\left(i\right)$ is composed of two degradation factors (i.e., nonlinearity and temperature variations): $\Delta RPR\left(i\right)\;=\;\left|\Delta RPR_{NL}\left(i\right)\right|\;+\;\left|\Delta RPR_{Temp}\left(i\right)\right|$. Therefore, the following condition needs to be satisfied for the correct recognition of the water level:

(10) $$\Delta RPR\left(i\right)\;=\;\left|\Delta RPR_{NL}\left(i\right)\right|\;+\;\left|\Delta RPR_{Temp}\left(i\right)\right|\;<\;\left|\Delta RPR_{ref}\right|$$

where $\Delta RPR_{ref}\;=\;\left(P_{signal}\left(0\right)\;-\;P_{signal}\left(N\right)\right)/\left(P_{ref}\cdot N\right)$ is defined as the reference RPR step.

**Figure 5:**
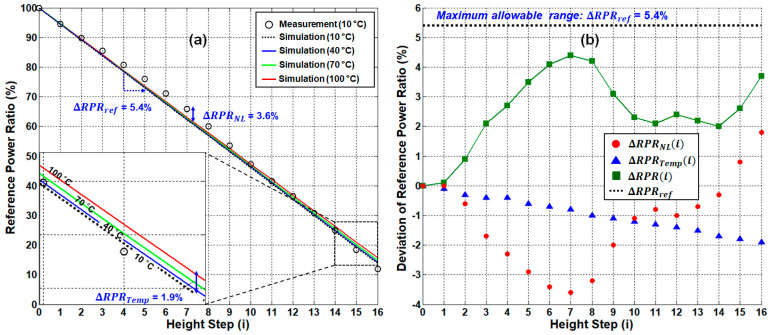
(**a**) Reference-to-power ratio (RPR) according to the water level in the back-to-back condition and (**b**) the RPR deviation according to the system performance degradation factors. The inset of Figure 5a shows the enlargement part of the RPR at the highest water level.

Based on Equations (9) and (10), we investigated the RPR performance of the sensing system in a back-to-back condition. Figure 5a shows the measured (hollow circles) and simulated (dotted and solid lines) RPR as a function of the water level. The RPR curve shows the linear response, with the negative slope where $\Delta RPR_{ref}$ is approximated to be 5.4% per step (see the dashed black line in Figure 5a). In addition, we investigated the impact of temperature variation in the water [21]. The simulation results reflecting the change in water temperature from 10 to 100 ℃ shows that the maximum value of $\left|\Delta RPR_{Temp}\left(i=16\right)\right|$ is less than 1.9%. It is worth noting that the higher the water level, the greater the variation in total received power.

Next, we analyzed $\Delta RPR\left(i\right)$ in comparison to $\left|\Delta RPR_{ref}\right|$. In Figure 5b, $\Delta RPR_{NL}\left(i\right)$ (see the red circles) is obtained from the actual measurement (being less than 3.6% at maximum) while $\Delta RPR_{Temp}\left(i\right)$ (see the blue triangles) is obtained from the simulation. Then, we obtain $\Delta RPR\left(i\right)$ (see the green rectangles) by adding the absolute values of each RPR deviation (i.e., $\left|\Delta RPR_{NL}\left(i\right)\right|\;+\;\left|\Delta RPR_{Temp}\left(i\right)\right|$), as Equation (10) explains. The maximum value of $\Delta RPR\left(i\right)$ was ~4.4% at i=7, which is below $\Delta RPR_{ref}$ (i.e., 5.4%). Thus, the system stays reliable during operation in the back-to-back condition.

## 4. Remote-Sensing Capability

### 4.1. Single-Path Network Architecture with Rayleigh Back-Scattering Effects

The irregular microscopic structure of the silica fiber generates Rayleigh scattering as well as attenuation [32]. In the OFS network based on the single-path configuration (see Figure 1), the remote-sensing distance is limited by the backward-traveling components of the Rayleigh-scattered ASE light in the SMF. The impacts of RBS at 1550 nm can be modeled as a function of the SMF length (D) as follows [37,38]:

(11) $$R_{RBS}\;\left[\mathrm{dB}\right]\;=\;\left\{\begin{array}{cc} -32-10\cdot log_{10}\left(\frac{20}{D}\right), & D<20\;\mathrm{km}\\ -32,\;\; & D\geq 20\;\mathrm{km} \end{array}\right.$$

where $R_{RBS}$ defines the ratio of the optical power of backscattered light to that of the ASE light injected into the SMF pool. When D < 20 km, $R_{RBS}$ increases with the distance. However, $R_{RBS}$ is saturated to −32 dB when D ≥ 20 km [38]. Based on Equation (11), the received RBS power ($P_{RBS}$) at the monitoring station can be expressed as [30]:

(12) $$P_{RBS}\;\left[W\right]\;=\;\int_{\lambda }^{\lambda +\Delta \lambda_{ch}}{\left|E_{ASE}\left(\lambda \right)\right|}^{2}\cdot 10^{\left(\frac{-2L_{OC}}{10}\right)}\cdot 10^{\left(\frac{R_{RBS}}{10}\right)}d\lambda .$$

Thus, the total received optical power ($P_{OPM2}$) at the OPM2 of the single-path OFS network will be:

(13) $$P_{OPM2}\;=\;P_{signal}\left(i\right)\;+\;\Delta P_{i}\;+\;P_{RBS}$$

The problem is that the signal power ($P_{Signal}$) at OPM2 is considerably small compared to that of the seeded ASE light (e.g., <−30 dB), being comparable to $P_{RBS}$, especially when SUs are in the water.

**Figure 6:**
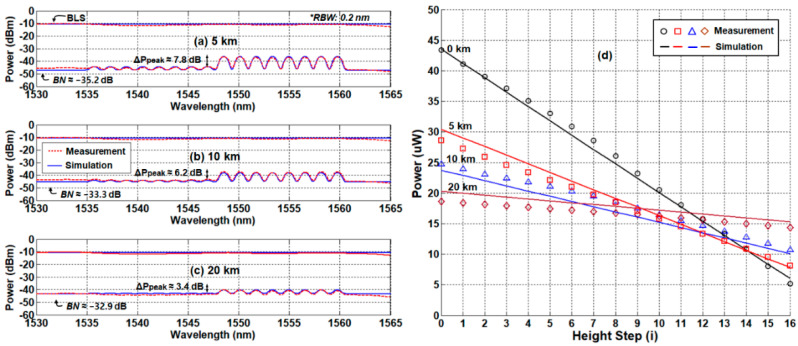
Simulation and measurement results at the different distances in the single-path configuration network. (**a**) The spectra of 1/2 water level at 5 km, (**b**) the spectra of 1/2 water level at 10 km, (**c**) the spectra of 1/2 water level at 20 km, and (**d**) optical-received power as a function of the water level.

We investigated the RBS effects via simulation as well as experiment. In the experiment, we used the standard SMF (ITU-T G.652.D) that induces the loss of 0.22 dB/km, indicating 0.44 dB/km attenuation for a round-trip. Figure 6a–c shows the received optical spectra in the half-full SFP case for three different distances (5 km, 10 km, and 20 km). This figure shows an increase in the background noise level and a decrease of the spectral peak power ratio ($\Delta P_{peak}$) when the sensing distance increases. Both experiment and simulation results imply that the background noise increases with SMF length due to RBS. As a result, the optical signal when SUs are in the water becomes indistinguishable from the background noise for an SMF length of > 5 km. This causes an increase in the offset of the received optical power versus water-level curves (see $P_{RBS}$ in Equation (11)). As a result, the received power at the highest water level (i.e., step 16) rises as the distance increases due to RBS, as seen in Figure 6d. Moreover, the slope of the curve decreases as the distance increases due to fiber-induced attenuation, e.g., 1.41 µW (5 km), 0.85 µW (10 km), and 0.31 µW (20 km). In other words, those two phenomena (the rise of offset and the decrease of slope) induce a decrease of dynamic range for the water-level measurement system, and it raises uncertainty, possibly providing false information. Here, the dynamic range is defined as the received optical power difference between the lowest (step 0) and highest water level (step 16).

### 4.2. Dual-Path Network Architecture for the Mitigation of the Rayleigh Back-Scattering Effect

**Figure 7:**
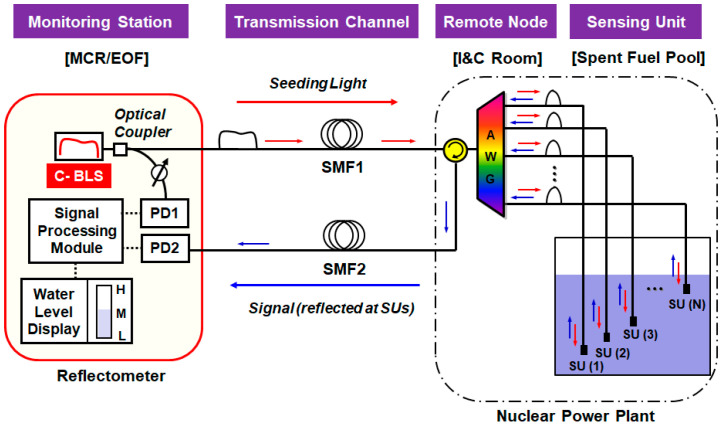
Architecture of the dense wavelength division multiplexing (DWDM)-passive optical fiber sensor network based on dual-path configuration.

Although the single-path DWDM-passive optical fiber sensor network has the advantage of simplicity, its remote-sensing distance is limited by RBS, as shown in Section 4.1. To sort it out, one can consider the dual-path architecture illustrated in Figure 7.

The dual-path OFS network utilizes two separate transmission paths: SMF1 for the transmission of BLS and SMF2 for the transmission of the optical signal from the remote node to the monitoring station. For this, we need to move the optical circulator from the monitoring station to the remote node at which the AWG is installed, still keeping the passive nature of the system. Then, the receiving part of the monitoring station does not suffer from RBS. Furthermore, the background noises (due to crosstalk and internal reflections on the network) are attenuated by the SMF2. For example, the background noise coefficient (BN) is estimated to be −49.5 dB, including the fiber attenuation of 8.8 dB (20 km × 0.22 dB/km × 2 times) at a 20 km distance. Thus, the total received optical power ($P_{OPM2}^{DP}$) at the monitoring station of the dual-path OFS network can be modeled as below:

(14) $$P_{OPM2}^{DP}\;=\;\int_{\lambda }^{\lambda +\Delta \lambda_{ch}}{\left|E_{ASE}\left(\lambda \right)\right|}^{2}\;\cdot \;\left[{\left|\sqrt{T_{AWG}\left(\lambda \right)}\right|}^{4}\;\cdot \;\gamma \left(i\right)\;\cdot \;10^{\left(-\frac{L_{IL}}{10}\right)}\;+\;10^{\left(\frac{BN-2L_{SMF}}{10}\right)}\right]d\lambda \;+\;\Delta P_{i}$$

**Figure 8:**
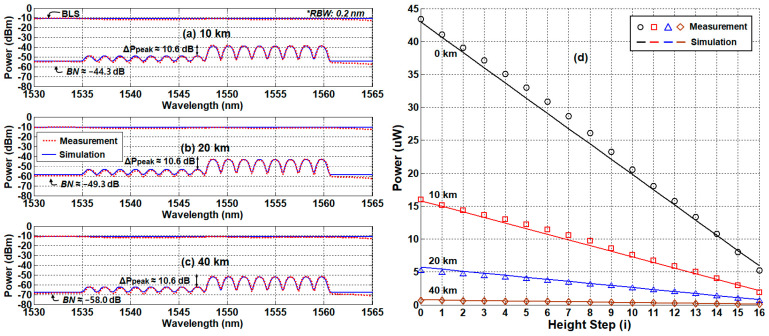
Measurement and simulation results at the different distances with dual-path network configuration. (**a**) The spectra of 1/2 water level at 10 km, (**b**) the spectra of 1/2 water level at 20 km, (**c**) the spectra of 1/2 water level at 40 km, and (**d**) optical-received power as a function of the water level with various fiber distances.

Figure 8a–c is the measured/simulated optical spectra of the received signal at three different distances (10 km, 20 km, and 40 km) that confirm two things we expected: (i) RBS is gone, and (ii) the background noise is further reduced due to the fiber attenuation. The spectral peak power ratio ($\Delta P_{peak}$) was maintained at 10.6 dB, while the sensing distance increases. This implies that the dynamic range can be maintained regardless of the sensing distance. As such, the SMF-induced signal attenuation remains the only factor that degrades the performance of the remote-sensing system. For qualitative verification, we measured the received optical power ($P_{OPM2}^{DP}$) as a function of water level for various distances (10 km, 20 km, and 40 km), Figure 8d. We also plotted the total received optical power of the back-to-back condition as a reference. Similar to the single-path network configuration, the total received optical power according to the water level decreases as the water level increases. In addition, the slope of $P_{OPM2}^{DP}$ decreases as the sensing distance increases due to the SMF loss. However, the offset of each curve is reduced, too, maintaining the dynamic range.

### 4.3. System Performance Comparison Between Single-Path and Dual-Path Network Configurations

**Figure 9:**
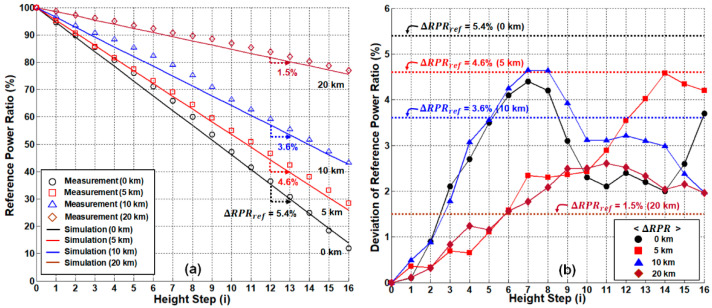
System performance of single-path network configuration. (**a**) Measured/simulated reference-to-power ratios according to the sensing distance. (**b**) Allowable deviation ranges of the reference-to-power ratios for remote sensing.

In this section, we compare the performance of two different network configurations (single-path and dual-path) based on RPR analysis, determining the maximum remote-sensing distance, respectively. First, we measure and calculate the RPR of the single-path network as a function of the water level for three different distances (5 km, 10 km, and 20 km), Figure 9a. For comparison, the back-to-back case is also plotted. Figure 9a shows that the slope of RPR curve gradually decreases as the distance increases due to the SMF-induced attenuation. In addition, the dynamic range of the RPR curve (i.e., $RPR\left(0\right)-RPR\left(16\right)$) further decreases as the SMF increases due to the effect of RBS (see Figure 6d).

Figure 9b shows ΔRPR for four different distances, where they are compared to $\Delta RPR_{ref}$ (revisit Section 3.3 for the theoretical background). For 5 km or less, ΔRPR was less than $\Delta RPR_{ref}$. However, when the distance was > 5 km (see 10 and 20 km), ΔRPR was larger than $\Delta RPR_{ref}$, indicating that the maximum sensing distance of the single-path OFS network is 5 km.

**Figure 10:**
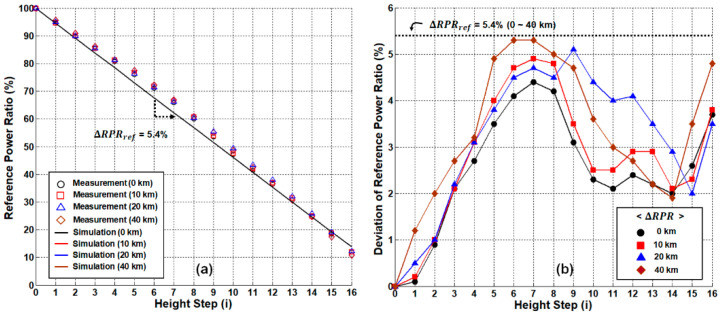
System performance of dual-path network configuration. (**a**) Measured/simulated reference-to-power ratios according to the sensing distance. (**b**) Allowable deviation ranges of the reference-to-power ratios for remote sensing.

Additionally, then, we investigated the system performance of the dual-path network configuration and compared it to the single-path results. Figure 10a shows the RPR curves of the dual-path case at different distances: 10 km, 20 km, and 40 km, respectively. Unlike the single-path case, the RPR curves do not significantly change regardless of the sensing distances. These results suggest two things: (i) the RBS effects are eliminated by the separation of the transmission channels, and (ii) there is no extra noise for the extended reach. In Figure 10b, we show ΔRPR for four different sensing distances (0 to 40 km), and compare them to $\Delta RPR_{ref}$. In all cases, ΔRPR was less than $\Delta RPR_{ref}$ (i.e., 5.4%), indicating that the dual-path network can provide the remote-sensing distance extended by >8 times as compared to the single-path network. In this case, the only limiting factor is the SMF loss that attenuates the power of the optical signal ($P_{OPM2}^{DP}$). Thus, the maximum distance is mainly determined by the sensitivity of OPM2. Note that the OPM’s typical sensitivity is ~1 nW, while the $P_{OPM2}$ is larger than 70 nW (at step 16) for the 40 km distance. Thus, the distance is expected to be up to 60 km.

## 5. Conclusions and Discussion

In this paper, we fully demonstrated the remote passive OFS network that utilizes simple optical power measurement. The OFS network is based on ASE light seeded to the remote node that comprises the AWG, where the ASE light is spectrum-sliced and distributed to multiple SUs. The SUs installed in the SPF (each SU is placed at a different height) back-reflect the incident light with different reflectivity, which is determined by the medium (i.e., the water vs. the air). The reflected lights from the multiple SUs are combined by the AWG, and then transmitted to the monitoring station. In the monitoring station, we utilized a simple OPM that includes the optical-to-electrical conversion devices (such as p-i-n photodiode) to obtain the water-level information. It subsequently could avoid the use of OSA, which is much more complex equipment than OPM, that is, reducing the cost, complexity, and processing time. However, the use of OPM required new criteria for the water gauging from the measured optical power. Thus, we proposed the new analysis process by using the RPR, which was obtained by dividing the received optical power by the reference optical power, which would be preset in the calibration process. However, when the SMF length was >5 km, the OFS network could not properly measure the water level due to the RBS at the fiber-optic cables. Thus, we separated the transmission channel into two different paths: one for delivering the ASE light from the monitoring station to the remote node, and the other for transporting the signals from the multiple SUs to the monitoring station. As a result, we could extend the sensing distance to >40 km.

In spite of various advantages of the proposed OFS network, the system performance can be degraded due to the floating particles (such as dust) or small particles dissolved in the water, which make the water turbid. This is because the proposed system is a sort of contact sensor, where the SUs may be stained with these particles. However, it can be mitigated considerably by the purification system of SFP. This purification function is carried out through one or two flow paths to prevent the corrosion of spent fuels and related facilities [30]. In addition, maintenance activities, such as a periodic performance test, are helpful to monitor the condition of the SUs with the optical spectrum analyzer.

Another issue to discuss is the temporal power fluctuation that would result in the water level misreading. This is mainly attributed to the optical power fluctuation caused by BLS. The output power instability of the used BLS was <±0.02 dB after 1 h warm-up time, and it caused the received optical power to be 5.22 ± 0.03 μW (indicating Δ*RPR* of 0.07%) when all SUs were immersed in the water (i.e., step 16) with B-t-B configuration. This variation value is negligible as compared to other degradation factors introduced in Section 3.3. However, in increasing the remote-sensing distance for the single-path configuration, the system would be more vulnerable to power fluctuation due to the RBS generated by the transmitted BLS. The maximum-allowable SMF length was only 5 km; thus, the power variation increased by about three times (<±0.1 μW, corresponding to Δ*RPR* of 0.3%) as compared to the B-t-B system. Otherwise, based on the dual-path configuration, we could extend the reach up to 40 km. In this case, the power fluctuation of the BLS output is further attenuated by the SMF, and thus, the background noise of the OPM would be the limiting factor. Therefore, when all SUs are in the water, the received optical power was 75 ± 2 nW, which corresponds to a Δ*RPR* of 0.29%.

Considering the explicit investigation results, we believe the proposed OFS network could be deployed as an auxiliary monitoring system for a spent fuel pool under an emergency situation in which the power supply to the remote location is not available.

## Figures and Tables

**Table 1:** Comparison of various multiplexed passive optical fiber sensor networks based on power measurement method for water-level monitoring.

Multiplexing Method	SDM ^1^	TDM ^2^	DWDM	DWDM
Optical Source and Interrogator	BLS + OPM	OTDR ^3^	BLS + OPM	BLS + OPM
Multiplexer/Demultiplexer	Optical Splitter	Optical Splitter	AWG	AWG
Type of SU	Fibers with flat-cleaved end facet	Specially fabricated fiber connector	Optical patch cord (SC ^4^ type)	Optical patch cord (LC ^5^ type)
Channel Numbers	12	3	11	16
Remote-sensing application	Short-range	Short and medium-range	Short-range	Medium and long-range
Self-referencing function	Not provided	Not provided	Not provided	Provided
Architecture type	Single-path	Single-path	Single-path	Dual-path
Channel scalability	Low	Low	High	High
Simplicity	Simple	Rather complex (needs the control of transmission delay)	Very simple	Very simple
Reference	[25]	[6]	[31]	Proposed

^1^ SDM: Space Division Multiplexing, ^2^ TDM: Time Division Multiplexing, ^3^ OTDR: Optical Time Domain Reflectometry, ^4^ SC: Square Connector, ^5^ LC: Lucent Connector.
